# Corporation as a Doctor

**Published:** 1912-04

**Authors:** 


### Corporation as a Doctor.

Proposed Law by Loomis is Very Quickly Knocked Out. Not at all Liked. All Senators Hasten to Disown Any Participation. Was Buffalo Measure. Concern Said to be Even Now a Going Business in This City. From *The Express* Bureau.

Albany, March 22.—A few days ago *The Express* noted the introduction by Senator Loomis of a bill perpetually incorporating the J. H. Dye Medical Company and conferring upon the corporation powers to practice medicine and surgery, to make medicines, to advertise remedies, to hold property, etc.

Today the bill was favorably reported by the judiciary committee and was immediately attacked by Hinman and Brackett and also by Wagner, the majority leader. The chairman of the judiciary committee washed his hands of the bill. The introducer attempted a defense, but the bill was sent to the committee of the whole which buries it.

Hinman could not understand how the bill had been advanced by unanimous consent upon its introduction. He said it was repugnant to him to think of the legislature incorporating a group of men, none of whom were physicians, to go into the doctoring business even though they hired licensed physicians.

Turning toward Loomis he said: "I do not know a single Tammany senator who would have risked his reputation in introducing a bill of this character."

It was stated in the argument that the would-be incorporators of the Dye company were a lawyer and a couple of clerks in his office.

Senator Bayne, chairman of judiciary, appeared nettled that the bill had been reported and he explained that the measure had reached the floor by a poll of the committee and not after proper consideration. "This sometimes happens," said he, "for some apparently good reason or because of the popularity of the introducer."

"I hope the senator does not mean to say that this bill was reported because of the popularity of the introducer," asked Big Tim Sullivan.

"I was not talking of any special bill," suavely responded Bayne, "but of conditions in general."

In the lower house the bill had equal success in making its silent way to a certain stage, having a place on the third reading calendar. Mr. MacGregor had it laid aside today.

---

The above is reproduced entire from the *Buffalo Express* of March 23, 1912. We need add nothing to the subject proper but we want to emphasize two things: 1. The medical profession should realize that, to a very large degree, it has the support and co-operation of the daily press and also of legislators. It is not only untrue but damaging to our future influence for good, to complain as some pessimists do, that we are handicapped by the indifference or open opposition of these great factors.

2. No better illustration could be found of the fact that different minds, especially under different training and habits, regard the same thing from very different view points. The impropriety of licensing a corporation to carry on the work of an individual, and work for which the individual has to show personal attainment and ability, would seem to be obvious to any trained legal mind—not to mention the well known attitude of the medical profession toward institutions of this sort. Yet the bill was introduced not by a mere politician but by a trained lawyer, of high standing; not by a Tammany senator, as in Senator Hinman's scathing comparison, but by one regarded as a reformer; not by an "ignorant foreigner"—who, by the way, seems to be rather a bogey than anything else—but by a man of culture and education whose claim to the term American is second only to the Indian's. In short, we have a bill that, according to the habits of thought of the medical profession, seems perfectly abominable, and yet introduced by a man who, in his private and professional life, is esteemed as an upright, honest, intelligent Christian gentleman. This incident will help us to realize how much allowance must be made for personal opinion and habit of thought, in securing the reforms that seem so urgent and so immediately necessary to us, and how patient we must be in our endeavors to make others think as we do.

